# CMTM3 as a Potential New Immune Checkpoint Regulator

**DOI:** 10.1155/2022/2103515

**Published:** 2022-09-20

**Authors:** Qian Shen, Zhirong Cong, Ying Zhou, Yue Teng, Jin Gao, Weiyan Tang

**Affiliations:** ^1^Department of Hematologic Lymphoma, Affiliated Cancer Hospital of Nantong University, Nantong, China; ^2^Department of Medical Oncology, Jiangsu Cancer Hospital and Jiangsu Institute of Cancer Research and The Affiliated Cancer Hospital of Nanjing Medical University, Nanjing, China

## Abstract

**Objectives:**

To evaluate the role of CKLF-like MARVEL transmembrane domain containing 3 (CMTM3) in tumor microenvironment and cancer immunotherapy and explore its potential mechanism.

**Method:**

The cancer genome map was obtained from the UCSC Xena database. RNAseq data from the Genotype-Tissue Expression (GTEx) and The Cancer Genome Atlas (TCGA) databases were utilized for evaluating the expression and prognostic value of CMTM3 through survival data of clinical trials. The enrichment analysis of CMTM3 was performed using the R package “clusterProfiler.” The scores of immune cell infiltration in TCGA samples were downloaded from the ImmuCellAI database and TIMER2 database, and the relationship between both immune cell invasion and CMTM3 expression was investigated. Immunological activation and suppression genes, immune checkpoints, chemokines, and their receptors were all investigated in relation to CMTM3.

**Results:**

Most tumor types had varied levels of CMTM3 expression and predicted poor survival status. The CMTM3 expression is closely associated with cancer-associated fibroblasts, macrophages, myeloid dendritic cells, endothelial cells, immune activation genes, immune suppressor genes, immune checkpoints, chemokines, and related receptors.

**Conclusion:**

Our data reveal that CMTM3 might be used as a cancer biomarker. CMTM3 may work in conjunction with other immunological checkpoints to alter the immune milieu, which could lead to the establishment of new immunotherapy medicines.

## 1. Introduction

The buildup of various genetic changes resulting in the development of different neoantigens on the surface of tumor cells is one of the most essential characteristics of cancer [1, 2]. Malignant cells often evolve to evade the attack by the immune system, which is the biggest hurdle in the immune therapies for cancer [3, 4]. Till now, a number of immune evading processes have been discovered with regard to immunotherapy, and the endogenous “immune checkpoints,” which regulate immune responses after antigen activation, are also expressed. Anticancer medicines based on immune checkpoint inhibitors, such as antiprogrammed cell death protein 1 (PD-1), antilymphocyte activation gene 3 (LAG3), and anticytotoxic T lymphocyte-associated protein 4, have been developed as a result of these discoveries (CTLA4) [5–7].

CKLF-like MARVEL transmembrane domain containing 3 (CMTM3) is the main member of the chemokine-like factor family (CKLFS) and is also one of the chemokine-like factor genes located in a cluster on chromosome 16q22 [8], which is differentially expressed in various human malignant tumors and is directly linked to the malignant phenotype of cells. However, its upstream target genes and their related molecular regulatory mechanisms are not clear. CMTM3 is expressed on immune cells such as follicular helper T cells, activated CD4 memory T cells, and CD8 T cells [9]. Through its interaction with C-C chemokine receptor 4 (CCR4), CMTM3 plays a role in the growth of arthritis [10]. Previous research has discovered that CMTM3 inhibits cell migration and invasion and is linked to a favorable outcome in gastric cancer [11]. CMTM3 can participate in EMT induction by inhibiting the JAK2/STAT3 signaling pathway and may have a strong influence on the metastasis of liver cancer [12]. CMTM3 decreases EGFR expression and EGF-mediated tumorigenicity by promoting Rab5 activity in gastric cancer [13]. These findings suggest that CMTM3 might be critical in the modulation of tumors and immune system diseases.

We explore the CMTM3 expression in a variety of cancers and its correlation with the prognosis of cancer patients. This research evaluates the link between CMTM3 and immune activation genes, immunosuppressive genes, immune cell infiltration scores, immune checkpoints, chemokines, and chemokine receptors. Our findings demonstrate the potential mechanisms by which CMTM3 affects tumor microenvironment and cancer immunotherapy. This research will shed light on the functional significance of CMTM3 in cancer.

## 2. Methods

### 2.1. Data Collection

The cancer genome map was obtained from the UCSC Xena database (https://xenabrowser.net/datapages/). The RNAseq data from the Genotype-Tissue Expression (GTEx) (https://commonfund.nih.gov/GTEx/) and The Cancer Genome Atlas (TCGA) (https://tcga-data.nci.nih.gov/) databases were utilized for evaluating the expression and the prognostic value of CMTM3 through survival data of clinical trials. Enrichment analysis of CMTM3 was performed using the *R* package “clusterProfiler.”

### 2.2. Immune Cell Infiltration Score

The ImmuCellAI database (http://bioinfo.life.hust.edu.cn/web/ImmuCellAI/) and TIMER2 database (http://timer.cistrome.org/) were used to obtain and analyze immune cell infiltration scores.

### 2.3. Prognosis Analysis

The overall survival (OS) of TCGA cohort patients was evaluated by the Kaplan–Meier analysis. The significance of CMTM3 in predicting OS, progression-free interval (PFI), and disease-specific survival (DSS) in cancer patients was assessed by univariate Cox regression analysis.

### 2.4. Gene Enrichment Analysis

The correlation of CMTM3 with genes was analyzed utilizing the TCGA data. The following settings were used to choose genes related with CMTM3 (p0.05) for gene set enrichment analysis (GSEA) using the R package “clusterProfiler”: minGSSize = 10, nPerm = 1000, and maxGSSize = 1000 are valid values. 0.05 is the cutoff *p* value [14].

### 2.5. Statistical Analysis

The data were analyzed and reported as mean ± SD (standard deviation). R.3.6.2 was availed for conducting the statistical analysis, and to examine the difference among all the groups, Student's *t*-test was utilized. *P* < 0.05 was defined as statistically significant.

## 3. Result

### 3.1. CMTM3 Expression in Cancers in the TCGA Dataset

**Figure 1:**
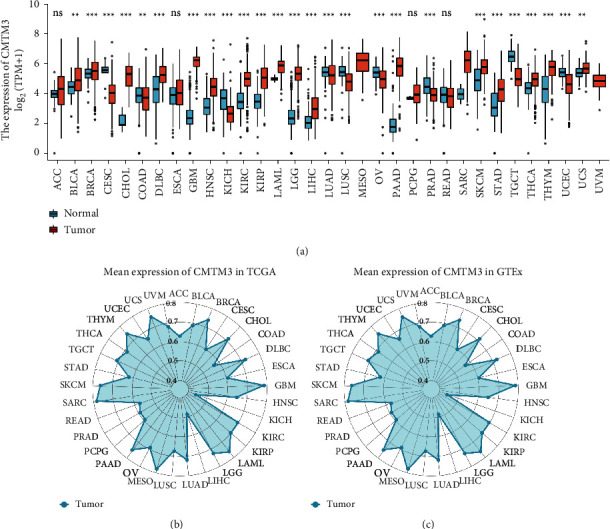
The CMTM3 expression in cancers. (a) The expression of CMTM3 between tumor tissues and normal tissues from the TCGA and GTEx database. (b) Data from the TCGA database on CMTM3 expression in tumor tissues. The mean value of the CMTM3 level is shown by the location of the dots. (c) From the GTEx database, CMTM3 expression in tumor tissues. The mean value of the CMTM3 level is shown by the location of the dots. ^*∗*^*P* < 0.05; ^*∗∗*^*p* < 0.01; ^*∗∗∗*^*p* < 0.001 and ^*∗∗∗∗*^*p* < 0.0001; ns, not significant.

The expressions of CMTM3 in cancers in the TCGA dataset were evaluated. The high expression and low expression of this molecule in different tumors were determined by the median of the tumor expression. The outcome showed that increased CMTM3 expression was observed in 21 types of tumors: ACC, BRCA, BLCA, CHOL, ESCA, DLBC, GBM, HNSC, KIRP, KIRC, LAML, LIHC, LGG, PAAD, PCPG, SKCM, SARC, STAD, THYM, THCA, and UCS. The low CMTM3 expression, on the other hand, was seen in ten different types of cancers: CESC, KICH, COAD, LUAD, LUSC, READ, OV, PRAD, TGCT, and UCEC (Figure 1(a)). Furthermore, CMTM3 was highly expressed in SARC, MESO, PAAD, LAML, GBM, BRCA, UCS, THYM, and SKCM in the tumor tissues of the TCGA dataset (Figure 1(b)). CMTM3 was highly expressed in SARC, MESO, LAML, GBM, DLBC, BRCA, USC, and SKCM in the tumor tissues of the GTEx dataset (Figure 1(c)).

The full names of tumors corresponding to all abbreviations in figures can be found in the TCGA database (https://tcga-data.nci.nih.gov/).

### 3.2. CMTM3 Expression in Tumors at Different WHO Stages

**Figure 2:**
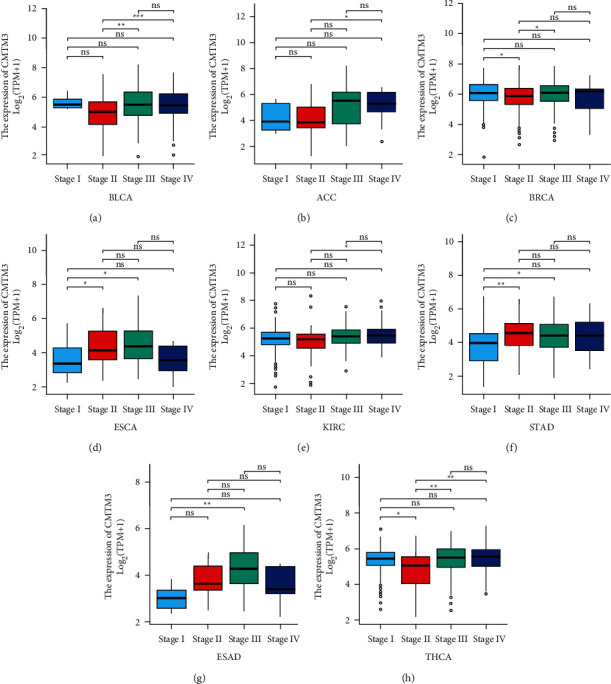
The expression of CMTM3 in tumors at different WHO stages. (a)–(h) The differential expression of CMTM3 in different tumor types at different WHO stages from the TCGA database. ^*∗*^*p* < 0.05; ^*∗∗*^*p* < 0.01; ^*∗∗∗*^*p* < 0.001 and ^*∗∗∗∗*^*p* < 0.0001; ns, not significant.

The CMTM3 expression in tumors at different WHO stages was further assessed. It was found that CMTM3 expression was low in the later stages of most tumors, including BLCA, BRCA, KIRC, STAD, and THCA (Figures 2(a), 2(c), 2(e), 2(f), 2(h)). Conversely, high CMTM3 expression was observed at earlier-stage tumors, including ACC, ESCA, and ESAD (Figures 2(b), 2(d), and 2(g)).

### 3.3. CMTM3 Expression in Different Tumors and Paired Paratumor Tissues

**Figure 3:**
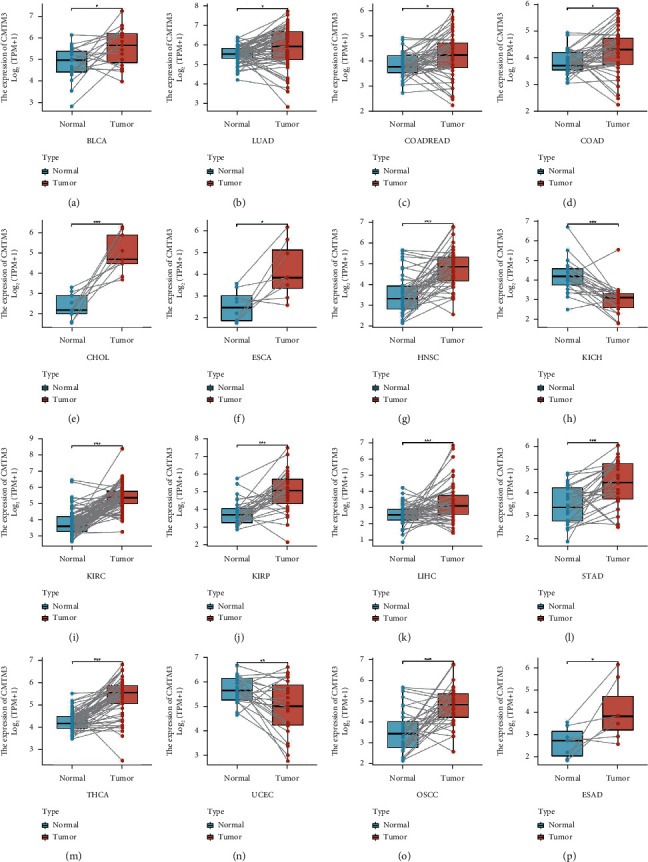
The expression of CMTM3 in various cancers. (a)–(p) CMTM3 expression in paired tumors and surrounding paratumor tissues in different cancer types identified from the TCGA database. ^*∗∗*^*P* < 0.01; ^*∗∗∗*^*p* < 0.001 and ^*∗∗∗∗*^*p* < 0.0001.

The CMTM3 expression was found to be medium to high in paired tumors and paratumor tissues in the TCGA database in BLCA, LUAD, COADREAD, COAD, CHOL, ESCA, HNSC, KIRC, KIRP, LIHC, STAD, THCA, OSCC, and ESAD, while low expression levels were found in KICH and UCEC (Figure 3).

The full names of tumors corresponding to all abbreviations in figures can be found in the TCGA database (https://tcga-data.nci.nih.gov/).

### 3.4. Prognostic Value of CMTM3

**Figure 4:**
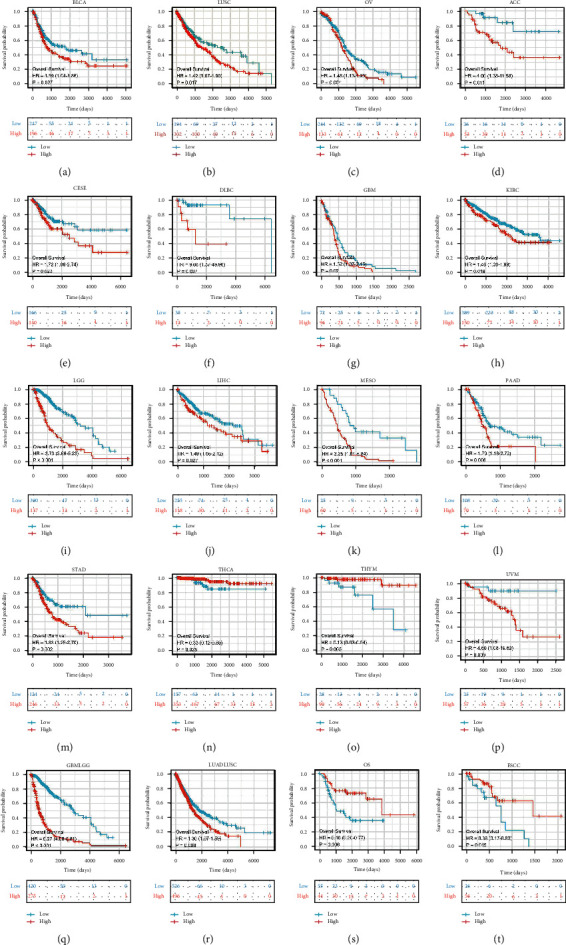
The expression of CMTM3 and overall survival analyzed by the Kaplan–Meier curves. (a)–(t) The expression of CMTM3 and Kaplan–Meier overall survival curves in the TCGA database for the specified tumor types. The break-off value for each tumor is the mean CMTM3.

**Figure 5:**
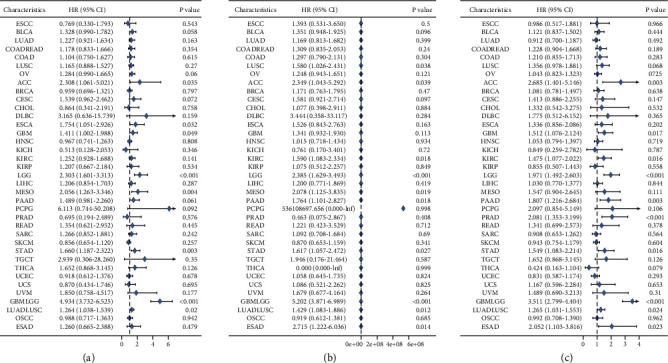
Forest maps on the expression of CMTM3 analyzed by univariate Cox regression. (a) CMTM3 for OS. (b) CMTM3 for DSS. (c) CMTM3 for PFI.

The prognostic value of CMTM3 in cancer patients was evaluated. Analysis of OS by Kaplan–Meier curves showed that CMTM3 was a safeguarding parameter in patients with BLCA, LUSC, OV, CESC, DLBC, ACC, GBM, LGG, KIRC, LIHC, PAAD, MESO, STAD, UVM, GBMLGG, and LUADLUSC THCA, and THYM and a risk factor in ESCC patients (Figure 4). The univariate Cox regression analysis on OS showed that CMTM3 was a protective factor in ESCC, CHOL, KICH, and PRAD patients, as well as BLCA, LUAD, COADREAD, COAD, OV, ACC, CESC, DLBC, KIRC, ESCA, KIRP, LGG, MESO, LIHC, PAAD, READ, PCPG, SARC, TGCT, STAD, THCA, UVM, and GBMLGG, and a risk factor in ESAD patients (Figure 5(a)). DSS analysis demonstrated that CMTM3 was a threatening element in PCPG patients only, and the difference was not statistically significant in other tumors (Figure 5(b)). Finally, PFI analysis demonstrates that CMTM3 is a protective factor in patients with LUAD, KICH, KIRP, SARC, SKCM, THCA, and UCEC, as well as in COADREAD, COAD, LUSC, ACC, CHOL, CESC, DLBC, GBM, ESCA, KIRC, LGG, MESO, PCPG, PAAD, PRAD, READ, TGCT, STAD, UVM, GBMLGG, and LUADLUSC and a threatening element in ESAD patients (Figure 5(c)).

### 3.5. GSEA Analysis of CMTM3

**Figure 6:**
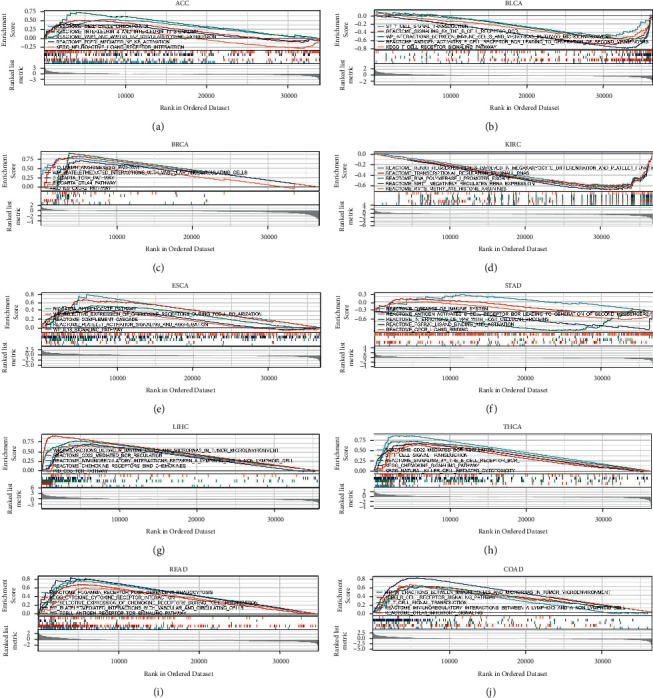
GSEA of CMTM3 in a variety of tumors. (a)–(j) The TOP20 GSEA terms in identified tumor types.

The pathways by which CMTM3 may be involved were assessed using the GSEA in thirty-three tumor types from the TCGA database. The outcomes suggest that CMTM3 may be connected to the pathways related to immune functions. These findings show that CMTM3 is involved in the regulation of the tumor immune microenvironment (Figures 6(a)–6(k)).

### 3.6. Analysis of Immune Cell Infiltration

**Figure 7:**
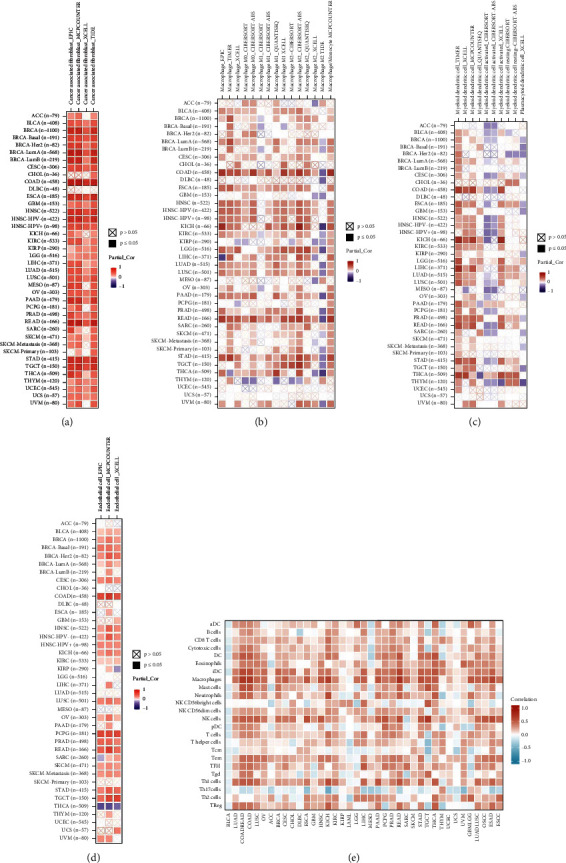
Analysis of tumor immune cell infiltration. (a)–(d) The relationship between CMTM3 and penetration of cancer-associated fibroblasts, macrophages, myeloid dendritic cells, and endothelial cells using the TIMER2 database. (e) Utilizing data from the ImmuCellAI database, the association between CMTM3 and infiltration of specified immune cells was investigated. ^*∗*^*P* < 0.05; ^*∗∗*^*p* < 0.01; ^*∗∗∗*^*p* < 0.001; and ^*∗∗∗∗*^*p* < 0.0001.

We used immune cell infiltration data from the TIMER2 database to conduct a correlation study to see if there was an association between CMTM3 expression and immune cell infiltration. In analyzing clinical tumor biopsies, tumor quality has a significant impact on the extraction of immune cell infiltrates. After making the necessary adjustments for tumor purity/quality, the CMTM3 expression was found to be considerably linked with different types of immune cells. The TIMER2 database results showed that CMTM3 was directly related to the invasion of cancer-associated fibroblasts, macrophages, myeloid dendritic cells, and endothelial cells in several TCGA tumor types (Figures 7(a)–7(d)). The correlation analysis by availing the data from the ImmuCellAI database demonstrated that CMTM3 was also directly proportional to the amount of infiltration of macrophages, dendritic cells, and others (Figure 7(e)). These cells are all important components of the tumor microenvironment (TME). The TME plays a critical role in the tumorigenesis, development, and metastasis of tumors. These results suggest that an increase in CMTM3 leads to an increase in cells such as cancer-associated fibroblasts, macrophages, myeloid dendritic cells, and endothelial cells, which can potentially modulate the protumor immune microenvironment to help tumors achieve immune escape. These factors are not conducive to improving the effectiveness of tumor immunotherapy, suggesting that CMTM3 plays the role of an “oncogene” in most tumors.

**Figure 8:**
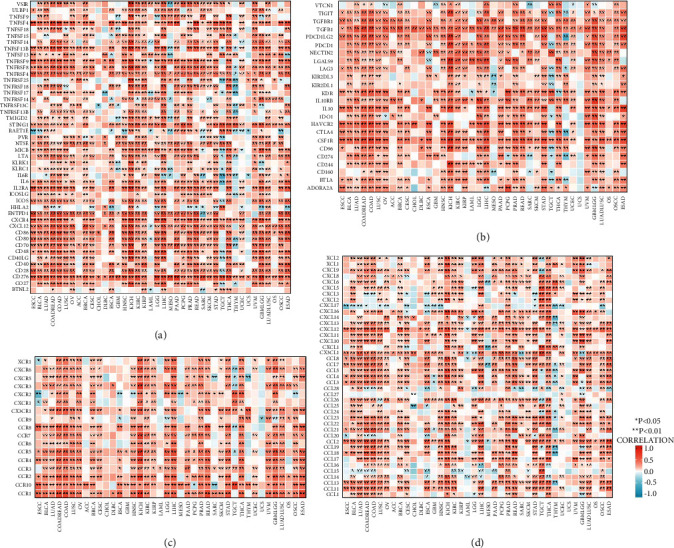
The heatmaps showing the correlation between CMTM3 and genes related to immunoregulation. (a) CMTM3 expression and activation genes for the immune system. (b) CMTM3 expression and immunosuppressive genes. (c) CMTM3 expression and chemokine receptor genes. (d) CMTM3 expression and chemokine genes. ^*∗*^*P* < 0.05; ^*∗∗*^*p* < 0.01; ^*∗∗∗*^*p* < 0.001; and ^*∗∗∗∗*^*p* < 0.0001.

Further research revealed that CMTM3 was directly connected to most of the immune activation genes, genes related to immune suppression status, chemokine receptor genes, and chemokine genes, and all of them are important elements of tumor microenvironment. The interaction of tumor cells with immune cells and surrounding tissues forms a complex integrated microenvironmental system. The existence of tumor microenvironment enhances tumor cell proliferation, migration, and immune evasion, which promotes tumor occurrence and development (Figures 8(a)–8(d)).

## 4. Discussion

Immune checkpoint inhibitors have transformed the face of cancer treatment in recent years, becoming a vital mode of immunotherapy for cancer treatment [15, 16]. Immune checkpoint inhibitors suppress immunity and induce a durable anticancer response [17–19]. Immune checkpoint proteins mainly include PD-L1, PD-1, and CTLA4 [20]. Previous studies have shown that CMTM3 can affect the efficacy of tumor immunotherapy by affecting the tumor microenvironment. The current research investigated the function of CMTM3 in various cancer types.

We evaluated the CMTM3 expression and its prognostic value in various types of cancers. We found that CMTM3 has high expression in 21 types of tumor tissues including BLCA, ACC, BRCA, DLBC, CHOL, ESCA, HNSC, GBM, KIRC, KIRP, LAML, LIHC, LGG, PAAD, PCPG, SKCM, SARC, STAD, THCA, THYM, and UCS and low expression in 10 types of tumor tissues including COAD, CESC, KICH, LUAD, OV, PRAD, LUSC, READ, TGCT, and UCEC. We then further assessed CMTM3 expression in tumors at separate WHO stages, and the outcome showed that its expression was low in most tumors at later stages, such as BLCA, BRCA, KIRC, STAD, and THCA. However, it was also observed in other tumors that CMTM3 has high expression in later stages, such as ACC, ESCA, and ESAD. For paired tumor and normal tissue analysis in TCGA tumors, we found that CMTM3 is highly expressed in BLCA, LUAD, COADREAD, COAD, CHOL, HNSC, KIRC, ESCA, KIRP, LIHC, STAD, THCA, OSaCC, and ESAD cancer tissue compared to normal tissue. However, CMTM3 was highly expressed in normal tissues rather than cancer tissue in KICH and UCEC.

OS analysis using Kaplan–Meier curves demonstrates that CMTM3 was a protective factor in patients with BLCA, LUSC, OV, CESC, ACC, DLBC, KIRC, GBM, LGG, LIHC, MESO, PAAD, UVM, STAD, GBMLGG, LUADL, and USC, while being a risk factor in THCA, THYM, OS, and ESCC patients. The univariate Cox regression analysis on OS showed that CMTM3 was a protective factor for ESCC, CHOL, KICH, and PRAD patients, while being a risk factor for BLCA, LUAD, COADREAD, COAD, OV, ACC, CESC, DLBC, KIRC, ESCA, KIRP, LIHC, LGG, MESO, PCPG, PAAD, READ, SARC, TGCT, STAD, THCA, UVM, GBMLGG, and ESAD patients. However, using OS as a clinical outcome may make clinical research less feasible, since deaths from noncancer causes do not always reflect tumor biology, aggressiveness, or response to therapy. Furthermore, utilizing OS frequently necessitates more follow-up time. As a result, the use of DSS or PFI in clinical studies can adequately reflect the influence of variables on patients. In light of these findings, we conducted a univariate Cox regression analysis to determine the relationship between CMTM3 and DSS or PFI in cancer patients. The DSS analysis showed that CMTM3 was only a risk factor in PCPG patients, and the difference was not statistically significant in other tumors. Finally, PFI analysis demonstrated that CMTM3 is a protective factor in patients with LUAD, KICH, KIRP, SARC, SKCM, THCA, and UCEC, as well as in COADREAD, COAD, LUSC, ACC, CESC, DLBC, CHOL, ESCA, KIRC, GBM, LGG, PAAD, PCPG, MESO, PRAD, STAD, TGCT, READ, UVM, GBMLGG, and LUADLUSC and a threatening element in patients with ESAD. These results suggest that the high CMTM3 expression is primarily a risk factor in most tumor types.

We further analyzed the signaling pathways by which CMTM3 may be involved using GSEA in 33 tumor types from the TCGA database. The results showed that CMTM3 was associated with immune-related pathways, such as the BIOCARTA_CTLA4_pathway, Reactomesignaling_by_the_B_cell_receptor_BCR, KEGG_T_cell_receptor_signaling_pathway, PID-IL8-cxcr2 pathway, PID_lymph_angiogenesis_pathway, and other pathways. These results indicate that CMTM3 plays a significant role in the regulation of tumor immune microenvironment.

Cancer cells are surrounded by an abundant matrix composed of complex extracellular matrix (ECM) proteins, including collagen and fibronectin, and numerous cellular components such as immune cells, endothelial cells, pericytes, and cancer-associated fibroblasts. The favorable interaction between tumor cells, matrix, and cells is critical for tumor formation, progression, and metastasis [21–23]. Results of immune cell infiltration analysis in our study showed that CMTM3 was favorably linked with the invasion of cancer-associated fibroblasts, macrophages, myeloid dendritic cells, and endothelial cells in most tumors.

It has also been reported that CALD1, a key gene associated with cancer-associated fibroblasts, can promote the progression of bladder cancer by remodeling the tumor microenvironment [24]. By modifying the tumor immune milieu, macrophages regulate malignant capabilities of colorectal cancer, according to Zhang et al. [25].

Miller et al. [26] believed that PD-L1 expression in tumor-associated dendritic cells in the tumor microenvironment was connected to improve survival in stage III colon cancer and could reflect an immunologically “hot” tumor microenvironment. Another study found that anlotinib modifies the tumor immune microenvironment by downregulating the expression of PD-L1 on vascular endothelial cells [27]. Therefore, our results suggest that an increase in CMTM3 leads to an increase in cancer-associated cells such as fibroblasts, macrophages, myeloid dendritic cells, and endothelial cells, which can potentially modulate the protumor immune microenvironment, thereby helping tumors achieving immune escape, which ultimately is not conducive to improving the effectiveness of tumor immunotherapy. These results are consistent with our previous pancancer analysis. The expression of this molecule in most cancer tissues is higher than that in adjacent tissues. Further research found that CMTM3 was positively correlated with most of the immune activation genes, genes related to immune suppression, chemokine receptor genes, and chemokine genes, which are all important components of the tumor microenvironment. The presence of tumor microenvironment enhances tumor cell proliferation, migration, and ability for immune escape, thereby promoting the occurrence and development of tumor. These findings support CMTM3's involvement as an immunological checkpoint regulator.

There are some limitations to this study. An in vivo experiment was not performed to test the antitumor activity of targeting CMTM3. More clinical trials need to be conducted to confirm the role of CMTM3 as an immune checkpoint regulator.

## 5. Conclusion

Taken together, we evaluated the role of CMTM3 as a potential prognostic indicator and its role in regulating tumor immunotherapy by affecting the tumor microenvironment. CMTM3 could be a target for tumor immunotherapy and a novel immune checkpoint regulator.

## Data Availability

The data used to support the findings of this study are available from the corresponding author upon request.

## References

[1] Dersh D., Phelan J. D., Gumina M. E. (2021). Genome-wide screens identify lineage- and tumor-specific genes modulating MHC-I- and MHC-II-restricted immunosurveillance of human lymphomas. *Immunity*.

[2] Church S. E., Galon J. (2015). Tumor microenvironment and immunotherapy: the whole picture is better than a glimpse. *Immunity*.

[3] Zeng D., Ye Z., Wu J. (2020). Macrophage correlates with immunophenotype and predicts anti-PD-L1 response of urothelial cancer. *Theranostics*.

[4] Cao R., Yang F., Ma S. C. (2020). Development and interpretation of a pathomics-based model for the prediction of microsatellite instability in Colorectal Cancer. *Theranostics*.

[5] Stephen T. L., Payne K. K., Chaurio R. A. (2017). SATB1 expression governs epigenetic repression of PD-1 in tumor-reactive T cells. *Immunity*.

[6] Zhao Y., Lee C. K., Lin C. H. (2019). PD-L1:CD80 Cis-Heterodimer triggers the Co-stimulatory receptor CD28 while repressing the inhibitory PD-1 and CTLA-4 pathways. *Immunity*.

[7] Turnis M. E., Sawant D. V., Szymczak-Workman A. L. (2016). Interleukin-35 limits anti-tumor immunity. *Immunity*.

[8] Zhou Z., Ma Z., Li Z. (2021). CMTM3 overexpression predicts poor survival and promotes proliferation and migration in pancreatic cancer. *Journal of Cancer*.

[9] Yu Z. L., Zhu Z. M. (2021). Comprehensive analysis of N6-methyladenosine -related long non-coding RNAs and immune cell infiltration in hepatocellular carcinoma. *Bioengineered*.

[10] Delic S., Thuy A., Schulze M. (2015). Systematic investigation of CMTM family genes suggests relevance to glioblastoma pathogenesis and CMTM1 and CMTM3 as priority targets. *Genes Chromosomes & Cancer*.

[11] Su Y., Lin Y., Zhang L. (2014). CMTM3 inhibits cell migration and invasion and correlates with favorable prognosis in gastric cancer. *Cancer Science*.

[12] Li W., Zhang S. (2017). CKLF-like MARVEL Transmembrane domain-containing member 3 (CMTM3) inhibits the proliferation and tumorigenisis in hepatocellular carcinoma cells. *Oncology Research Featuring Preclinical and Clinical Cancer Therapeutics*.

[13] Yuan W., Liu B., Wang X. (2017). CMTM3 decreases EGFR expression and EGF-mediated tumorigenicity by promoting Rab5 activity in gastric cancer. *Cancer Letters*.

[14] Thorsson V., Gibbs D. L., Brown S. D. (2018). The immune landscape of cancer. *Immunity*.

[15] Yoo M. J., Long B., Brady W. J., Holian A., Sudhir A., Gottlieb M. (2021). Immune checkpoint inhibitors: an emergency medicine focused review. *The American Journal of Emergency Medicine*.

[16] Wladis E. J., Kambam M. L. (2022). Ophthalmic complications of immune checkpoint inhibitors. *Orbit*.

[17] Lisi L., Lacal P. M., Martire M., Navarra P., Graziani G. (2022). Clinical experience with CTLA-4 blockade for cancer immunotherapy: from the monospecific monoclonal antibody ipilimumab to probodies and bispecific molecules targeting the tumor microenvironment. *Pharmacological Research*.

[18] Cremolini C., Vitale E., Rastaldo R., Giachino C. (2021). Advanced nanotechnology for enhancing immune checkpoint blockade therapy. *Nanomaterials*.

[19] Li Y., Liu J., Gao L. (2020). Targeting the tumor microenvironment to overcome immune checkpoint blockade therapy resistance. *Immunology Letters*.

[20] Xu J., Brosseau J. P., Shi H. (2020). Targeted degradation of immune checkpoint proteins: emerging strategies for cancer immunotherapy. *Oncogene*.

[21] Simon T., Gagliano T., Giamas G. (2017). Direct effects of anti-angiogenic therapies on tumor cells: VEGF signaling. *Trends in Molecular Medicine*.

[22] Liao Z., Tan Z. W., Zhu P., Tan N. S. (2019). Cancer-associated fibroblasts in tumor microenvironment - accomplices in tumor malignancy. *Cellular Immunology*.

[23] Brandao M., Simon T., Critchley G., Giamas G. (2019). Astrocytes, the rising stars of the glioblastoma microenvironment. *Glia*.

[24] Du Y., Jiang X., Wang B. (2021). The cancer-associated fibroblasts related gene CALD1 is a prognostic biomarker and correlated with immune infiltration in bladder cancer. *Cancer Cell International*.

[25] Zhang Y., Zhao Y., Li Q., Wang Y. (2021). Macrophages, as a promising strategy to targeted treatment for colorectal cancer metastasis in tumor immune microenvironment. *Frontiers in Immunology*.

[26] Miller T. J., Anyaegbu C. C., Lee-Pullen T. F., Spalding L. J., Platell C. F., McCoy M. J. (2021). PD-L1+ dendritic cells in the tumor microenvironment correlate with good prognosis and CD8+ T cell infiltration in colon cancer. *Cancer Science*.

[27] Liu S., Qin T., Liu Z. (2020). Anlotinib alters tumor immune microenvironment by downregulating PD-L1 expression on vascular endothelial cells. *Cell Death & Disease*.

